# Vectorial Electron Spin Filtering by an All-Chiral Metal–Molecule Heterostructure

**DOI:** 10.1021/acs.jpclett.2c00983

**Published:** 2022-06-30

**Authors:** Chetana Badala Viswanatha, Johannes Stöckl, Benito Arnoldi, Sebastian Becker, Martin Aeschlimann, Benjamin Stadtmüller

**Affiliations:** † Department of Physics and Research Center OPTIMAS, University of Kaiserslautern, Erwin-Schrödinger-Straße 46, 67663 Kaiserslautern, Germany; ‡ Department of Chemistry, University of Kaiserslautern, Erwin-Schrödinger-Straße 52, 67663 Kaiserslautern, Germany; § Institute of Physics, Johannes Gutenberg University Mainz, Staudingerweg 7, 55128 Mainz, Germany

## Abstract

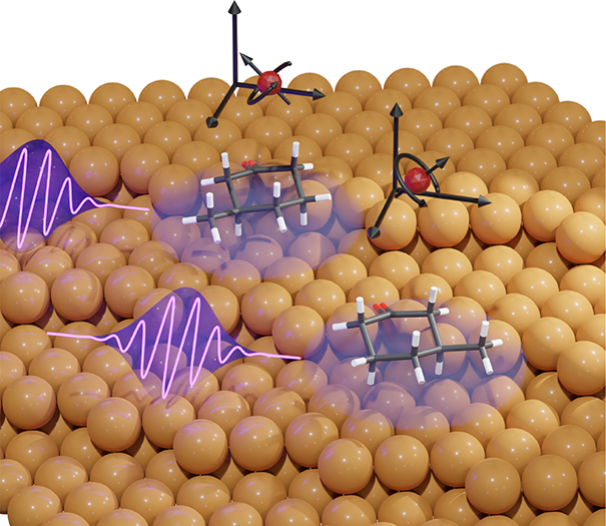

The discovery of the electrons’ chiral induced spin selective transmission (CISS) through chiral molecules has opened the pathway for manipulating spin transport in nonmagnetic structures on the nanoscale. CISS has predominantly been explored in structurally helical molecules on surfaces, where the spin selectivity affects only the spin polarization of the electrons along their direction of propagation. Here, we demonstrate a spin selective electron transmission for the point-chiral molecule 3-methylcyclohexanone (3-MCHO) adsorbed on the chiral Cu(643)^R^ surface. Using spin- and momentum-resolved photoelectron spectroscopy, we detect a spin-dependent electron transmission through a single layer of 3-MCHO molecules that depends on all three components of the electrons’ spin. Crucially, exchanging the enantiomers alters the electrons’ spin component oriented parallel to the terraces of the Cu(643)^R^ surface. The findings are attributed to the enantiomer-specific adsorption configuration on the surface. This opens the intriguing opportunity to selectively tune CISS by the enantiospecific molecule–surface interaction in all-chiral heterostructures.

Chirality^1,2^ is a highly intriguing and ubiquitous phenomenon in nature with severe implications for biology, biochemistry, and pharmacology. It describes the incongruency of an object with its mirror image and is most famously observed in (bio)molecules such as amino acids and sugars. Chiral materials exist in the left- and right-handed forms called *S* and *R* enantiomers, which both reveal identical physical properties as long as they do not interact with other chiral objects. In contact with other chiral materials, however, they can reveal distinct enantiomer selectivity that is today most frequently employed in drug development.^3^

Crucially, chirality and chirality-induced functionalities are not only limited to molecular systems but also can be found in crystals, at surfaces, or in other low-dimensional adsorbate systems. For instance, high Miller index surface planes of otherwise achiral metal crystals reveal kink sites that are inherently chiral.^4^ This surface chirality can, for instance, lead to enantiospecific adsorption of chiral molecules on chiral surfaces.^5,6^ Chiral interaction at surfaces is also of fundamental interest for addressing the origin of homochirality in biomolecules that remains unanswered.^7,8^

One of the most famous chirality-induced surface functionalities is the chiral induced spin selectivity (CISS) effect^9,10^ with a vast range of applications in spintronics,^11,12^ enantioselective chemical reactions,^13,14^ and biological electron transfer.^15^ The CISS effect describes the spin selective transmission of electrons through a layer or thin film of oriented chiral molecules grown on a surface.^16,17^ It has been predominantly investigated for molecules on noble metals, although recent studies have explored this phenomenon also in more complex materials such as hybrid organic–inorganic perovskites.^18−20^ For the more conventional case of chiral molecules on metal surfaces, CISS has been demonstrated almost exclusively for structurally helical molecules such as helicenes^21,22^ or in helical polymers such as DNA^23,24^ or oligopeptides.^25^ In these systems, the sign of the spin polarization of the transmitted electrons is determined by only the winding direction of the molecular helix, while the substrate itself acts as only a template for orienting the helical molecular axis along the surface normal.^26^ In this upright standing geometry, spin selective transmission can be observed only for the longitudinal (out-of-plane) spin component of the electrons along the direction of their propagation through the molecular layer. This unidirectional or scalar spin filtering in structurally helical molecules severely limits the range of application of the CISS effect, for instance, for manipulating spin currents in spintronics applications.

One strategy for overcoming this severe limitation of structurally helical molecules is to employ molecules with different types of chirality. The first indications of the feasibility of this approach were demonstrated by Niño et al.^27^ They experimentally observed different transmissions of the longitudinal and the transversal spin component of polarized electrons from a ferromagnetic cobalt surface passing through chiral molecules that are not structurally helical.^27^ While this study provided a first glimpse of the CISS effect in structurally nonhelical molecules on surfaces, many fundamentals such as the microscopic spin filtering mechanism or the orientation of the spin selectivity axis of structurally nonhelical molecules on nonmagnetic surfaces are largely unexplored.

In this Letter, we shine new light on the CISS effect in point-chiral molecules on surfaces using spin- and angle-resolved photoemission spectroscopy. This experimental approach allows us to directly quantify the polarization of the initially unpolarized electrons of the surface after transmission through the chiral molecules grown on top. As a model system, we have selected the chiral 3-methylcyclohexanone (3-MCHO) enantiomers adsorbed with submonolayer coverage on a naturally chiral Cu(643)^R^ surface. Such a combination of a chiral molecular adsorbate and a chiral surface has so far not been explored in CISS studies despite its unique ability to control the molecular adsorbate structure by the enantiospecific molecule–surface interaction. For the structurally well-characterized all-chiral model system 3-MCHO on Cu(643)^R^,^28−34^ we will provide evidence for the existence of CISS that leads to a spin filtering of electrons depending on all three components of the electrons’ spin. Exchanging the enantiomers inverts the sign of the spin polarization only for the component of the three-dimensional (3D) spin vector that is oriented parallel to the terraces of the naturally chiral Cu(643)^R^ surface while leaving the other components unaffected. Our findings demonstrate the potential to manipulate the 3D spin-dependent transmission in point-chiral molecules by the enantiospecific molecule–surface interaction, and the resulting enantiospecific adsorption configuration in all chiral metal–molecule heterostructures.

We start our discussion with the structural properties of the adsorbate system. A structural model of the ideally terminated Cu(643)^R^ surface and the enantiomers of 3-MCHO are shown in Figure 1a. The naturally chiral Cu(643)^R^ surface was prepared by repeated cycles of argon ion sputtering and sample annealing at 1000 K for 30 min. While thermal annealing reduces the number of kinks of the surface, the net surface chirality is still retained.^32^ For such a well-annealed surface, the average terrace width is ≈7.8 ± 0.3 Å^31^ and the steps are oriented ∼19° from the [110] close-packed direction.^31^ Moreover, the chiral kink sites are found in the [1̅32̅] surface direction. A more detailed explanation of the miscuts and the corresponding crystallographic directions can be found in the literature.^35,36^

**Figure 1:**
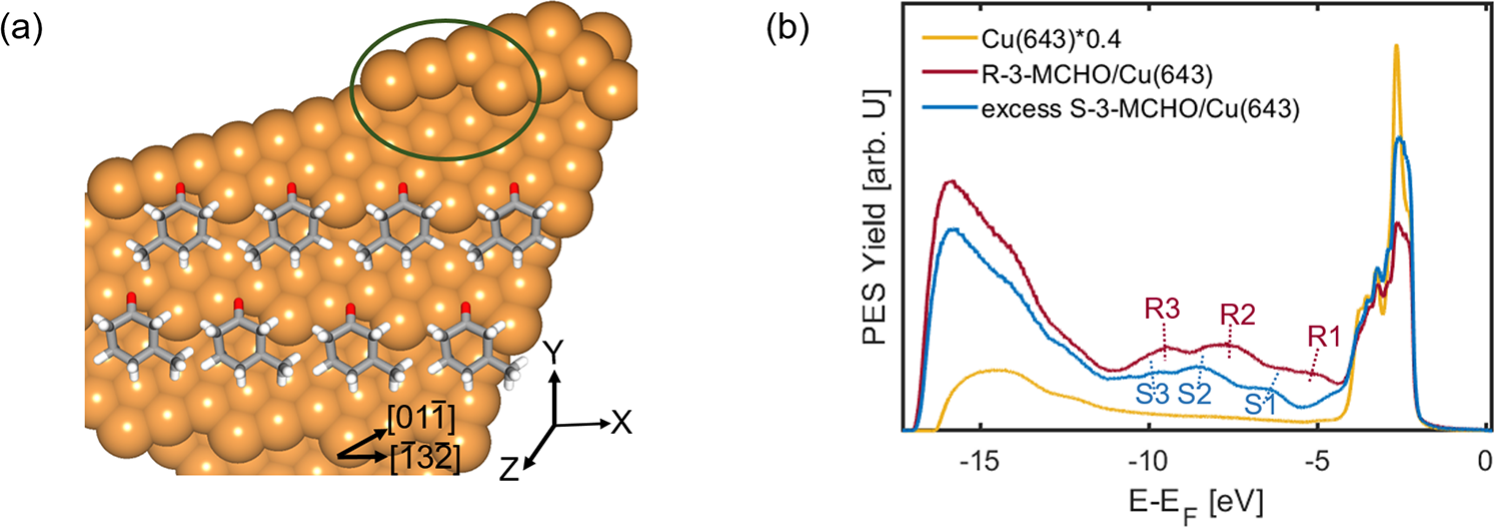
(a) Model depicting the ideal Cu(643)^R^ surface and the *R* and *S* enantiomers of 3-MCHO. The green ellipse shows a vacant chiral kink site. R-3-MCHO and S-3-MCHO are shown adsorbed on the upper and lower terrace, respectively, to depict enantiospecific adsorption of the two enantiomers. (b) Valence band spectra recorded for the surface normal of (111) terraces (*h*ν = 21.2 eV).

The 3-MCHO submonolayer films were prepared from the commercially available pure *R* enantiomer and from a racemic mixture of the *R* and *S* enantiomers. The racemic mixture had to be used because no pure *S* enantiomers are commercially available and the enantiomer separation of the *S* enantiomer is challenging, as for most chiral molecules with C, H, and O atoms. Instead, the *S* enantiomer-dominated films were prepared from the racemic mixture following the enantioselective kinetic separation procedure described by Horvath et al.^37^ This procedure results in only *S* enantiomer-dominated films when using the Cu(643)^R^ surface, and not for the Cu(643)^S^ surface. This inherent consequence of the enantioselective kinetic separation procedure necessitates the use of the Cu(643)^R^ surface in our study. At room temperature, these sample preparation procedures result in submonolayer coverages of the *R* and *S* enantiomers on the surface that predominantly adsorb at kink sites of the Cu(643)^R^ surface^29^ as illustrated in Figure 1a. The kink site- and enantiomer-specific adsorption of the 3-MCHO molecules results in highly ordered molecular films with distinct adsorption configurations of both enantiomers. This allows us to correlate the experimentally observed spin-dependent electron transmission to the geometry and crystallographic directions of the all-chiral metal–molecule heterostructure.

The first crucial step of our study is the confirmation of the successful preparation of submonolayer films of the *R* and *S* enantiomers on Cu(643)^R^. We employ ultraviolet photoelectron spectroscopy to study the characteristic spectroscopic signatures of the different interfacial states using He I_α_ radiation (21.2 eV) and normal emission with respect to the (111) terraces. The corresponding valence band structures of the substrate and both enantiomers on Cu(643)^R^ are shown in Figure 1b.

The valence band structure of the bare Cu(643)^R^ surface (orange solid line) is dominated by the strong emission features of the Cu 3d bands in the energy range between *E* – *E*_F_ values of −2 and −4 eV. After the adsorption of both enantiomers, the intensity of the Cu d-bands is severely reduced and new characteristic molecular features appear in the energy range between −5 and −10 eV. These features are labeled as R1–R3 for R-3-MCHO and S1–S3 for the S-3-MCHO-dominated molecular film and appear at different binding energies. These different characteristic binding energies for both samples confirm the formation of a different metal–molecule interface and the successful preparation of an S-3-MCHO-dominated film on the Cu(643)^R^ surface from the racemic mixture. In fact, the absence of any spectral signature of the molecular orbitals of R-3-MCHO in the valence band spectrum of the S-3-MCHO-dominated film suggests an extremely high concentration of S-3-MCHO enantiomers on the surface that exceeds by far the lower limit of 75% determined by Horvath et al.^37^ For the sake of simplicity, we will refer to this system as S-3-MCHO even though it is not strictly speaking a pure S-3-MCHO film on the Cu(643)^R^ surface. In addition, the energetic differences of the molecular valence states for the enantiomers can be attributed to different enantiospecific adsorption energies of both 3-MCHO enantiomers at kink sites of the Cu(643)^R^ surface.^30,33,34^ They also point to different enantiospecific deformations and bond pairs,^38^ which already suggests different adsorption geometries of the *R* and *S* enantiomers on the surface.

To quantify the spin-dependent transmission of photoelectrons through both 3-MCHO enantiomers, we record spin-resolved photoelectron spectra of the bare and the enantiomer-covered Cu(643)^R^ surface in the energy region of the Cu sp-bands between *E* – *E*_F_ ≈ −1 eV and the Fermi energy (*E* – *E*_F_ = 0 eV). This energy region was selected due to the absence of any spectroscopic signatures of molecular orbitals and allows us to study the intrinsic spin filtering of the CISS effect that is determined by the chirality of the total electron density of the molecule. In our experiment, we employ a new type of 3D spin analyzer. After the energy filtering in a conventional hemispherical electron spectrometer, the spin-polarized photoelectrons first pass a magnetic lens, the so-called spin rotator,^39^ before entering the FERRUM spin filter^40^ (FOCUS GmbH). This novel experimental scheme allows us to detect the longitudinal (out-of-plane) spin polarization *P*_*z*_ as well as both in-plane spin polarizations *P*_*x*_ and *P_y_* and hence to access the 3D spin vector of the photoemitted electrons. A more detailed description of the experimental setup can be found in the Experimental Methods and in the literature.^41^ With this tool in hand, we are able, for the first time, to fully quantify the spin selective electron transmission depending on the vectorial spin of the electrons and to fully characterize the orientation of the spin-sensitivity axis of a point-chiral molecule in 3D space.

We start with the photoemission yield and the corresponding spin polarization of the emitted electrons of the bare Cu(643)^R^ surface. The energy- and momentum-resolved photoemission yield is shown in Figure 2a. It is dominated by a parabolic feature that can be attributed to the Shockley surface state of the naturally chiral surface. Interestingly, the surface state parabola is centered at the Γ̅-point of the (643) miscut plane and not that of the (111) plane of the surface terraces. This suggests that the true normal emission geometry for this surface is the [643] direction. The spin polarization of the emitted electrons of bare Cu(643)^R^ can be fully characterized by the spin-resolved photoemission yield recorded for all three spin components, i.e., the longitudinal out-of-plane spin component *z* as well as both in-plane spin components *x* and *y*. In this coordinate system, the *x*-direction is parallel to the Cu(643)^R^ step edges and the terraces while the *y*-direction is oriented perpendicular to them. The corresponding three sets of spectra are shown in Figure 2b. The spectral yield of the respective collinear spin orientations (spin-up and spin-down) for each component is shown as red and blue solid lines. The characteristic maximum in all spectra at *E* – *E*_F_ = −0.25 eV can be attributed to the spectral yield of the Shockley surface state. The energy range below the surface state corresponds to the sp-band region. The most important observation is that the spin-up and spin-down spectra are coincident for all three components in the energy range of the sp-bands. This observation indicates a neglectable spin polarization of the photoemitted electrons of the Cu(643)^R^ surface in the sp-band region in normal emission geometry. This is further confirmed by the energy-resolved spin polarization of all three spin components, which is defined as the normalized difference between spin-up and -down electrons:
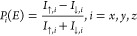
1

**Figure 2:**
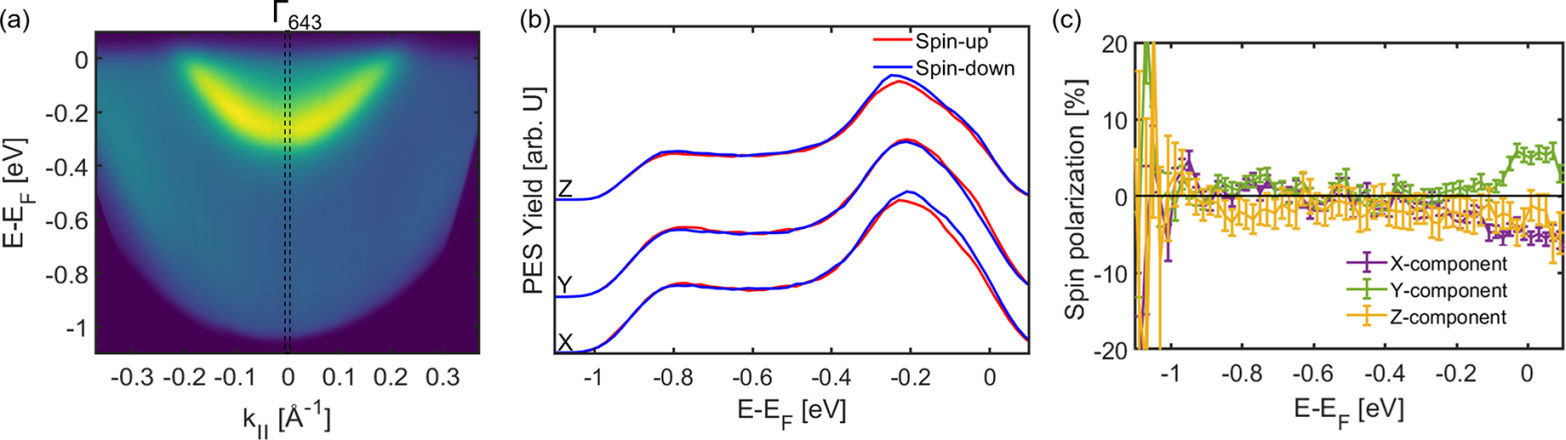
(a) Energy vs momentum photoemission intensity map showing the surface state of Cu(643)^R^. (b) Spin-resolved spectra of the bare Cu(643)^R^ surface measured along the dotted lines in panel a. (c) Corresponding spin polarizations for the spectra in panel b (*h*ν = 5.9 eV; p-polarized).

Within the experimental uncertainty, the spin polarization is zero for all energies smaller than *E* – *E*_F_ = −0.2 eV and all spin components as shown in Figure 2c. The non-zero polarization in the binding energy range between −0.2 and 0 eV is attributed to a residual spin polarization of the surface state of the Cu(643)^R^ surface at the Γ-point. Hence, we can conclude that any detected spin polarization of the 3-MCHO/Cu(643)^R^ interface for energies below −0.2 eV in normal emission geometry can directly be attributed to the spin-dependent electron transmission through the layer of chiral molecules.

The same set of spin-resolved photoemission experiments was repeated for both enantiomers on the surface. The spin-resolved photoemission spectra for all three vectorial spin components are shown in panels a and c of Figure 3, and the corresponding energy-resolved spin polarizations in panels b and d of Figure 3. All changes in the spectral line shape can be attributed to an attenuation of the photoemission signal of bare Cu(643)^R^. In particular, no additional molecular feature can be detected in this specific binding energy range, as expected from the valence band photoemission spectra (see Figure 1b and the Supporting Information). In contrast to the bare Cu(643)^R^ surface, we observe a significant spin polarization for all three vectorial components in the binding energy range of the Cu sp-bands (*E* – *E*_F_ < −0.2 eV) after the adsorption of both enantiomers. This points to a spin selective transmission of electrons through the 3-MCHO layer that depends on all three components of the electrons’ spin. Interestingly, only the sign of the *x*-component (purple data points) of the 3D spin vector changes when exchanging the enantiomers on the surface, while the spin polarization of the *y*- and *z*-components (green and yellow data points, respectively) remains almost identical. An alternative presentation of the spin polarizations for each spin component is shown in the Supporting Information. Hence, switching the enantiomers inverts only the spin polarization in the direction parallel to the terraces of the Cu(643)^R^ surface. This corresponds, in the first approximation, almost to a mirroring of the spin-sensitivity axis on the *y*–*z* plane of the surface.

**Figure 3:**
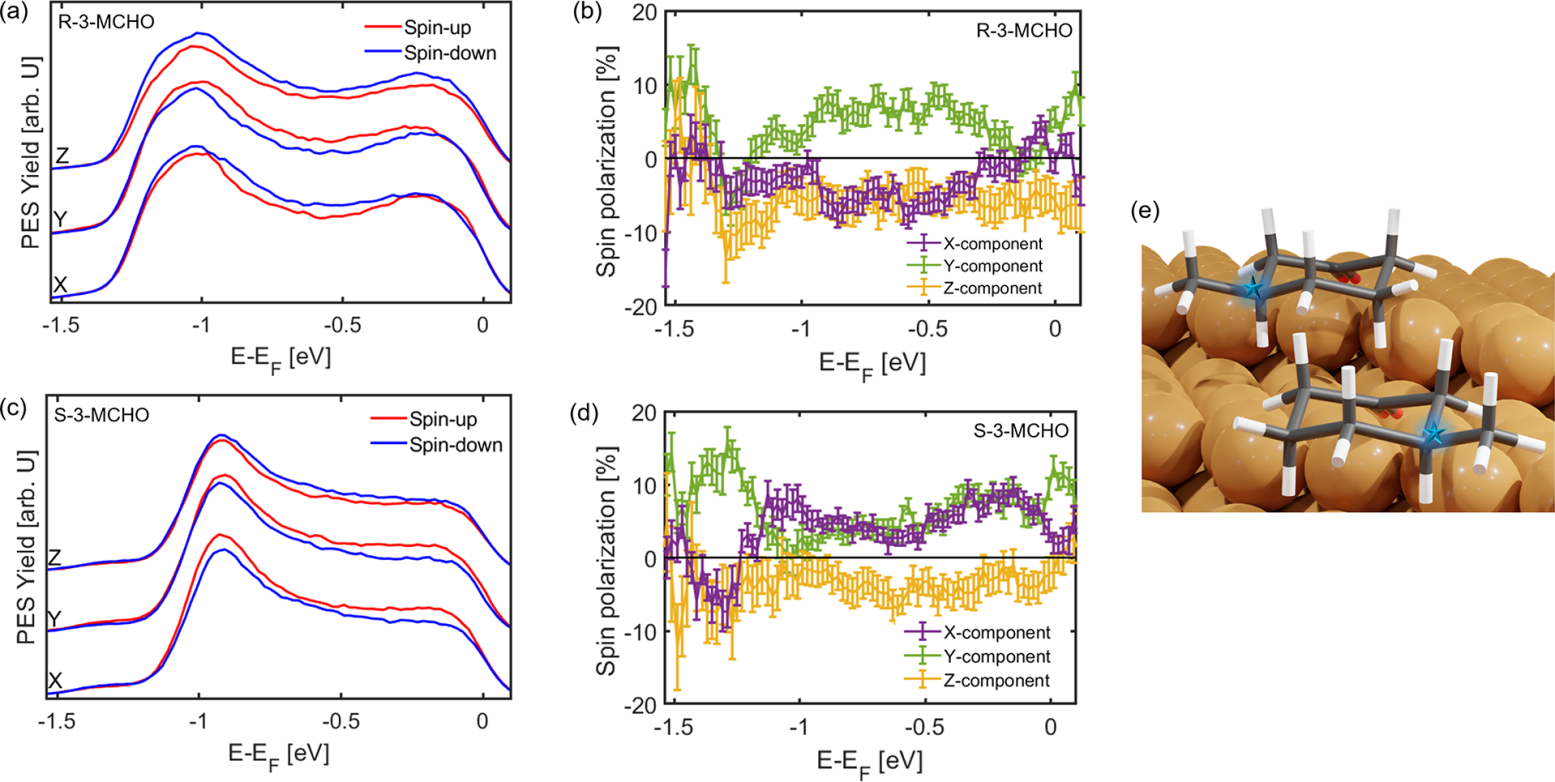
Spin-resolved photoemission spectra along the Cartesian coordinate axes of the manipulator where the sample is mounted for (a) R-3-MCHO/Cu(643)^R^ and (c) S-3-MCHO/Cu(643)^R^ (*h*ν = 5.9 eV; p-polarized). Corresponding spin polarizations for (b) R-3-MCHO/Cu(643)^R^ and (d) S-3-MCHO/Cu(643)^R^ (*h*ν = 5.9 eV; p-polarized). (e) Chair conformations with the chiral center marked by a blue star for R-3-MCHO (top) and S-3-MCHO (bottom).

This observation can be rationalized by the combination of the chiral molecular structure of both enantiomers themselves (shown in Figure 3e) as well as their adsorption geometry on the surface. In general, chiral molecules can exhibit diverse structural arrangements, but their overall molecular electron cloud exhibits either left- or right-handed^42^ chirality. This helical electron cloud that exists even for point-chiral molecules^43^ is responsible for the spin filtering effect of the 3-MCHO enantiomers, very similar to the CISS effect in prototypical structurally helical molecules such as helicene^21^ or DNA-like polymers.^25^ This is supported by recent vector-based theoretical studies that examined the chirality–helicity equivalence in *R* and *S* stereoisomers.^44^ Hence, the CISS effect in free-standing layers of structurally helical and point-chiral molecules is expected to be very similar.

In our case, however, we also have to consider the enantiomer-specific adsorption and the resulting adsorption geometries of the point-chiral 3-MCHO enantiomers on the surface. The enantioselective adsorption is largely attributed to steric effects, i.e., to the structural arrangement of the molecular groups,^45^ although theoretical predictions have also pointed out the decisive role of the helicities of the electron cloud for the enantiorecognition between the substrate and molecules.^46,47^ As the concept of helical matching in asymmetric catalysis^48^ has already been elucidated, we suppose that such helicity matching also plays a role in the enantioselective adsorption of chiral molecules on chiral surfaces. The adsorption geometry, on the contrary, is fully attributed to a steric effect. Previous studies have predicted a chair conformation for the cyclohexane ring with a tilt toward the methyl group and the side with the methyl group leaning toward the Cu(111) terrace. In this configuration, the oxygen atom and the C=O group are bound in the kink sites^30^ and the straight step edge while the cyclohexane ring tilts away from the kink site. At room temperature, the methyl group is present predominantly in the equatorial position (∼95%),^49^ and there are two enantiotopic positions with respect to the C=O group for the methyl group to occupy, which gives rise to the different enantiomers. Therefore, it is only logical to conclude that one enantiotopic position is located closer to the kink atoms than the other. In either case, the chiral carbon is on the terrace with the helicity^47^ surrounding it. This adsorption geometry explains the observation of the sign of the spin polarization along the direction of the terraces, i.e., the *x*-direction.

In conclusion, our work demonstrated the general nature of the CISS effect even for point-chiral molecules on nonmagnetic surfaces. In particular, we have shown a spin selective electron transmission for the point-chiral molecules 3-MCHO adsorbed on the naturally chiral Cu(643)^R^ surface. Unpolarized electrons from the substrate’s sp-band pass through the chiral molecule layer and exhibit spin polarizations that depend on all three components of the electrons’ spin (i.e., the spin selectivity is not just limited to the longitudinal out-of-plane direction as known for structurally helical molecules). Substituting one 3-MCHO enantiomer with its mirror image alters the spin-dependent transmission only for electrons with their spin component oriented along the terraces of the Cu(643)^R^ surface. This observation is rationalized by the characteristic enantiomer-specific adsorption configuration of both enantiomers on the surface that can exist for only the unique combination of chiral molecules and chiral surfaces. This opens the intriguing opportunity to selectively tune CISS by the enantiospecific molecule–surface interaction in all chiral heterostructures. In addition, our investigation also underlines the key role of chiral interactions in enantiorecognition and may explain the discrepancies that occur when only steric effects are taken into account.

## Experimental Methods

The spin- and momentum-resolved photoemission experiments were conducted with a hemispherical analyzer (SPECS Phoibos 150) that is equipped with both a CCD detector system and the commercial spin detector (Focus FERRUM^40^) that is mounted in a 90° geometry after the hemispherical analyzer’s exit slit plane. The FERRUM detector in combination with a spin rotator lens allows us to record spin-resolved photoemission data for three orthogonal spin components in this geometry that can be converted into two spin components parallel to the surface plane (in-plane spin components) and the out-of-plane spin component along the surface normal. The spin sensitivity or Sherman function (*S*) of this very-low-energy electron diffraction (VLEED) detector was determined to be 0.29 for all three spin components. As excitation sources, we use the monochromatic He I_α_ radiation (21.2 eV) of a high-flux He discharge source as well as the fourth harmonic of a Ti:sapphire laser oscillator (Tsunami long pulse, Ti:sapphire oscillator system) with a photon energy of 5.9 eV. In our experiments, we use p-polarized laser light, an angle of incidence of ≈45°, and a sample bias of −4 V for the laser experiments. More experimental details can be found in the Supporting Information.

## Supporting Information Available

Experimental details for the sample preparation of submonolayer films, supporting results of (spin-resolved) valence band photoemission spectroscopy, and further information regarding the 3D spin detection (PDF)
