# Supplementary material for: Vectorial Electron Spin Filtering by an All-Chiral Metal–Molecule Heterostructure
Source: J Phys Chem Lett. 2022 Jun 30;13(26):6244–9. doi: 10.1021/acs.jpclett.2c00983 (PMC9272820; doi:10.1021/acs.jpclett.2c00983)
Supplement: Supplementary file 1 — jz2c00983_si_001.pdf [file jz2c00983_si_001.pdf]

Vectorial Electron Spin Filtering by an All-Chiral Metal-Molecule Heterostructure

Chetana Badala Viswanatha[a], Johannes Stöckl[a], Benito Arnoldi[a], Sebastian Becker[a,b], Martin Aeschlimann[a], and Benjamin Stadtmüller*[a,c]

[a]Department of Physics and Research Center OPTIMAS, University of Kaiserslautern, Erwin-Schrödinger-Straße 46, 67663 Kaiserslautern, Germany.
[b]Department of Chemistry, University of Kaiserslautern, Erwin-Schrödinger-Straße 52, 67663 Kaiserslautern, Germany.
[c]Institute of Physics, Johannes Gutenberg University Mainz, Staudingerweg 7, 55128 Mainz, Germany
*bstadtmueller@physik.uni-kl.de

Sample Preparation of the Molecular Layers

The ketones, 3-Methylcyclohexanone (purity 97% , CAS Number: 591-24-2) and (R)-(+)-3-Methylcyclohexanone (purity 98%, CAS Number: 13368-65-5), were purchased from Sigma-Aldrich. The ketones were purified by subjecting them to several cycles of freezing, pumping and thawing before use to remove any high vapour pressure contaminants.[1]

In order to adsorb an excess of S enantiomer on the Cu(643)^R surface, we follow an enantioselective kinetic separation procedure described in literature.[2] The exposure of Cu(643)^R to racemic 3-MCHO followed by an annealing step leaves an excess of S-3-MCHO on the surface with an enantiomeric excess of at least 50 %. All experiments were performed at room temperatures, where the molecular adsorption is predominantly at kink sites.[3]

Valence Band Photoemission Spectroscopy of the Molecular Films

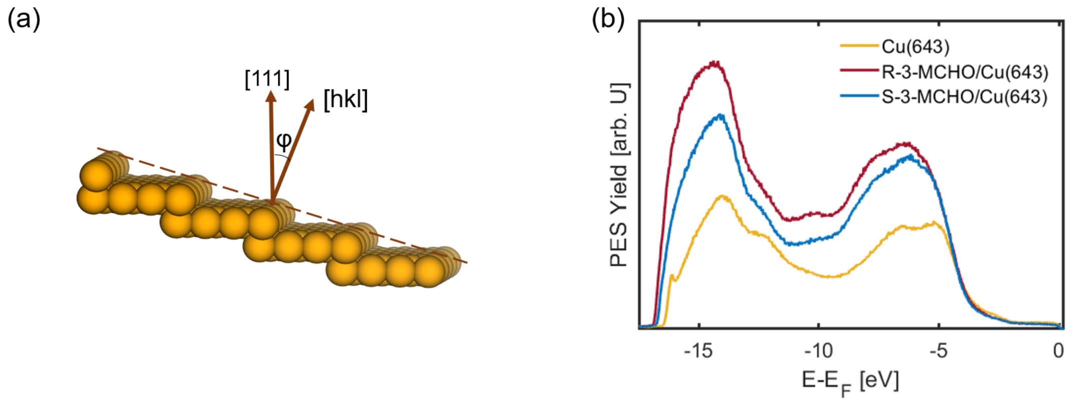

Figure S1. (a) A simplified model depicting the different surface normals for a vicinal crystal with (111) terraces. (b) UPS spectra at the surface normal of (643) surface (hν=21.2 eV).

A simplified model for vicinal crystals, representing the surface normals with respect to the (111) terraces and the optical surface, is shown in Figure S1(a). The angle ϕ between the [643] and [111] surface normals is $\sim$16°. In our experiment, the average step direction for the Cu(643)^R crystal was determined using the spot splittings in the LEED pattern.The convention for the nomenclature of Cu(643) surfaces and corresponding LEED patterns can be found in literature.[1]

A 6-axis manipulator positions the sample in the x-,y- and z-direction with a precision of 10 µm and allows sample rotations around the three axes with a precision up to 0.05°, which was used to access the different surface normals.

Figure S1(b) shows ultraviolet photoemission spectra (UPS) for the substrate and enantiomers adsorbed on the surface measured with He I_α radiation (21.2 eV) in the normal emission of the (643) plane. No additional molecular feature can be detected in the energy range -1.5 and 0 eV which is investigated in our laser experiments (hν=5.9 eV) .

3D Spin Detection

The spin-resolved measurements for each enantiomer/substrate system and the bare substrate were obtained for three orthogonal directions to get the full vectorial information.

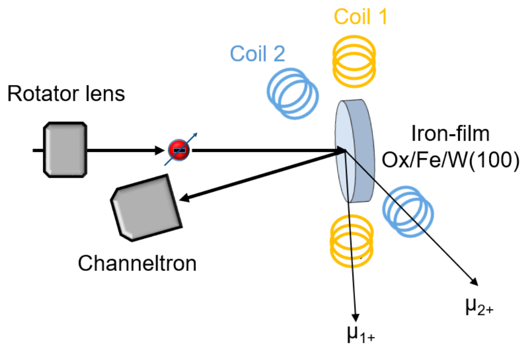

Figure S2. A schematic of the FERRUM detector with rotator lens

The FERRUM[4] detector uses two sets of magnetic coils to magnetize the oxidized iron scattering target. Figure S2 illustrates our experimental spin detection scheme. In our experimental geometry, the orientations of these coils allow us to record the component of the electron spin along the direction of the optical axis of the analyzer (out of plane direction for normal emission geometry of the sample) as well as the component of the electron's spin in the direction of the entrance slit of the analyzer (one in-plane direction for normal emission geometry of the sample). The second in-plane component can, in general, be recorded by using the spin rotator.[5] In our experiment, however, we record both in-plane spin components by using the spin rotator with rotation set as -45° and +45°. This allows for an almost identical alignment of the electron beam for both settings of the spin rotator, which also focuses the electron beam depending on the magnitude of the spin rotation.

The magnetization coils, C1 and C2, are sensitive in x-direction and z-direction, respectively. The rotator rotates the spin of the photoelectrons around the z-axis by +45°(R+) and -45° (R-). This enables the detection of spin in three orthogonal directions (C1R+, C1R- and C2). The magnetization direction is switched between scans[6] to account for laser intensity fluctuations.

The transformation of the data into the coordinate system of the sample surface is done using a rotation matrix, and the relation between the two coordinate systems is given below.

$$\begin{pmatrix} A_X \\ A_Y \\ A_Z \end{pmatrix} = \begin{pmatrix} \frac{1}{\sqrt{2}} & \frac{1}{\sqrt{2}} & 0 \\ -\frac{1}{\sqrt{2}} & \frac{1}{\sqrt{2}} & 0 \\ 0 & 0 & 1 \end{pmatrix} \begin{pmatrix} A_{C1R-} \\ A_{C1R+} \\ A_{C2} \end{pmatrix} \tag{S1}$$

Alternative View of the Spin-polarization for 3-MCHO Enantiomers

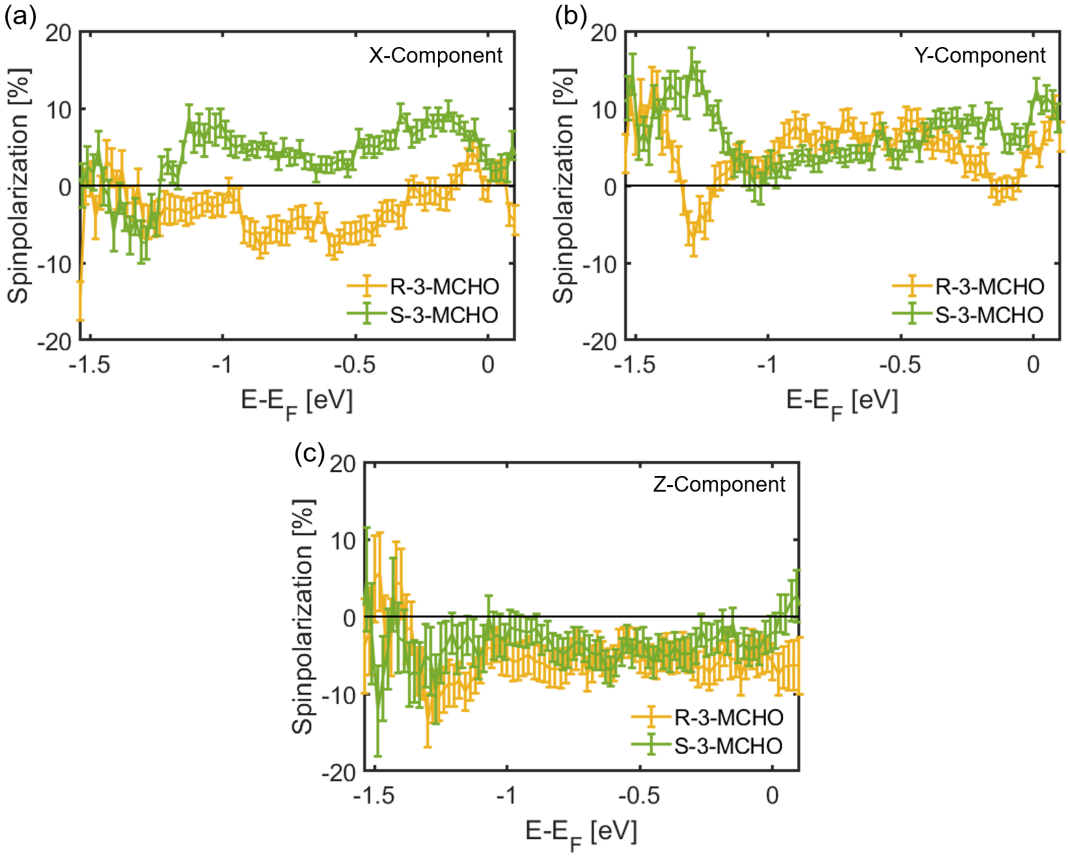

Figure S3. (a) X-component of the spin-polarization for the enantiomers of 3-MCHO on Cu(643)^R surface. (b) Y-component of the spin-polarization for the enantiomers of 3-MCHO on Cu(643)^R surface. (c) Z-component of the spin-polarization for the enantiomers of 3-MCHO on Cu(643)^R surface.

Figure S3 provides an alternative view of the energy-resolved spin-polarization for the R- and S-enantiomers of 3-MCHO on the Cu(643)^R surface.

References

(1) Gellman, A. J.; Horvath, J. D.; Buelow, M. T. Chiral Single Crystal Surface Chemistry. *J. Mol. Catal. A: Chem.* **2001**, *167*, 3–11.

(2) Horvath, J. D.; Koritnik, A.; Kamakoti, P.; Sholl, D. S.; Gellman, A. J. Enantioselective Separation on a Naturally Chiral Surface. *J. Am. Chem. Soc.* **2004**, *126*, 14988–14994.

(3) Horvath, J. D.; Gellman, A. J. Enantiospecific Desorption of Chiral Compounds from Chiral Cu(643) and Achiral Cu(111) Surfaces. *J. Am. Chem. Soc.* **2002**, *124*, 2384–2392.

(4) Escher, M.; Weber, N. B.; Merkel, M.; Plucinski, L.; Schneider, C. M. FERRUM: A New Highly Efficient Spin Detector for Electron Spectroscopy. *e-J. Surf. Sci. Nanotechnol.* **2011**, *9*, 340–343.

(5) Engwall, D. A.; Dunham, B. M.; Cardman, L. S.; Heddle, D. P.; Sinclair, C. K. A Spin Manipulator for Electron Accelerators. *Nucl. Instrum. Methods Phys. Res. A: Accel. Spectrom. Detect. Assoc. Equip* **1993**, *324*, 409–420.

(6) Winkelmann, A.; Hartung, D.; Engelhard, H.; Chiang, C.-T.; Kirschner, J. High Efficiency Electron Spin Polarization Analyzer Based on Exchange Scattering at Fe/W(001). *Rev. Sci. Instrum.* **2008**, *79*, 083303.
